# Unraveling the origin of ferroelectric resistance switching through the interfacial engineering of layered ferroelectric-metal junctions

**DOI:** 10.1038/s41467-021-27617-6

**Published:** 2021-12-15

**Authors:** Fei Xue, Xin He, Yinchang Ma, Dongxing Zheng, Chenhui Zhang, Lain-Jong Li, Jr-Hau He, Bin Yu, Xixiang Zhang

**Affiliations:** 1 Hangzhou Global Scientific and Technological Innovation Centre, Zhejiang University, Hangzhou, 311200 China; 2 Physical Science and Engineering Division, King Abdullah University of Science and Technology, Thuwal, 23955-6900 Saudi Arabia; 3 Department of Materials Science and Engineering, City University of Hong Kong, Kowloon, Hong Kong China

**Keywords:** Electrical and electronic engineering, Ferroelectrics and multiferroics

## Abstract

Ferroelectric memristors have found extensive applications as a type of nonvolatile resistance switching memories in information storage, neuromorphic computing, and image recognition. Their resistance switching mechanisms are phenomenally postulated as the modulation of carrier transport by polarization control over Schottky barriers. However, for over a decade, obtaining direct, comprehensive experimental evidence has remained scarce. Here, we report an approach to experimentally demonstrate the origin of ferroelectric resistance switching using planar van der Waals ferroelectric α-In_2_Se_3_ memristors. Through rational interfacial engineering, their initial Schottky barrier heights and polarization screening charges at both terminals can be delicately manipulated. This enables us to find that ferroelectric resistance switching is determined by three independent variables: ferroelectric polarization, Schottky barrier variation, and initial barrier height, as opposed to the generally reported explanation. Inspired by these findings, we demonstrate volatile and nonvolatile ferroelectric memristors with large on/off ratios above 10^4^. Our work can be extended to other planar long-channel and vertical ultrashort-channel ferroelectric memristors to reveal their ferroelectric resistance switching regimes and improve their performances.

## Abstract (web summary)

The mechanism of ferroelectric resistance switching is still under debate. Here, the authors propose an interfacial engineering approach to demonstrate its origin and find that it is governed by three independent variables: polarization, barrier change, and initial barrier.

## Introduction

Ferroelectric memory devices, which generally encode information through the reversal of ferroelectric polarization (i.e., ferroelectric domains), have found promising applications in next-generation computational technology, including neuromorphic computing and in-memory computing^1–4^. These devices feature high operation speed, excellent endurance/retention, and low-power dissipation. Among them, ferroelectric memristors, which have received considerable attention over the past decade^5–13^, are typically nonvolatile resistance switching devices with a simple two-terminal metal-ferroelectric-metal architecture. Based on their carrier transport mechanisms, ferroelectric memristors can be classified into ultrashort-channel and long-channel devices.

Ultrashort-channel ferroelectric memristors (channel length: a few nanometers) can be exemplified by vertical ferroelectric tunnel junctions, in which electron tunnel transmission primarily dominates carrier transport^14–17^. Theoretically, their ferroelectric resistance switching (FRS) regime has been proposed as the tuning of electron tunnel transmission by ferroelectric polarization-controlled barrier profile. However, previous studies have merely provided experimental evidence of either polarization evolution^5^ or barrier profile engineering^17,18^, rather than all FRS elements covering both domain configurations and the barrier profile. Moreover, owing to the presence of nonferroelectric origins, such as vacancy migration and redox across the ferroelectric channel^19,20^, the ultrashort-channel device regime remains debated thus far.

By contrast, owing to the channel length being far greater than electron screening length (e.g., twenty nanometers), long-channel ferroelectric memristors cannot be fully depleted by interior free carriers (given that the ferroelectric channel has a semiconducting property). The related electrical transport is, therefore, normally regulated by thermionic emission, instead of electron tunneling^14^. Based on this theory, a plausible hypothesis for understanding FRS is the modulation of thermionic emission by a ferroelectric polarization-dominated change in interface barrier^8,21–24^. Although this hypothesis is in part analogous to that in ultrashort-channel memristors, the carrier transport across the two types of devices fundamentally distinguishes their corresponding FRS phenomena. So far, for long-channel ferroelectric memristors, a comprehensive, experimental examination of their FRS operation regime is still lacking.

Here, we report an interfacial engineering approach to exploring the origin of FRS systematically and experimentally by combining ferroelectric domain mapping and electrical hysteresis probing. Emerging van der Waals (vdW) ferroelectric α-In_2_Se_3_ was used as a model material to create a planar long-channel device with two terminals. This not only facilitates in situ domain visualization, but also enables us to finely engineer charged interfacial molecules and Schottky barriers at both terminals. Three independent variables, i.e., ferroelectric polarization, polarization-charge-induced barrier changes, and initial Schottky barriers, are found to produce FRS and optimize the memristor on/off ratio. Based on these findings, a volatile ferroelectric memristor and a nonvolatile device with over 10^4^ on/off ratio are realized. Our approach is also applicable for ultrashort-channel ferroelectric memristors to demonstrate all FRS elements.

## Results

**Fig. 1:**
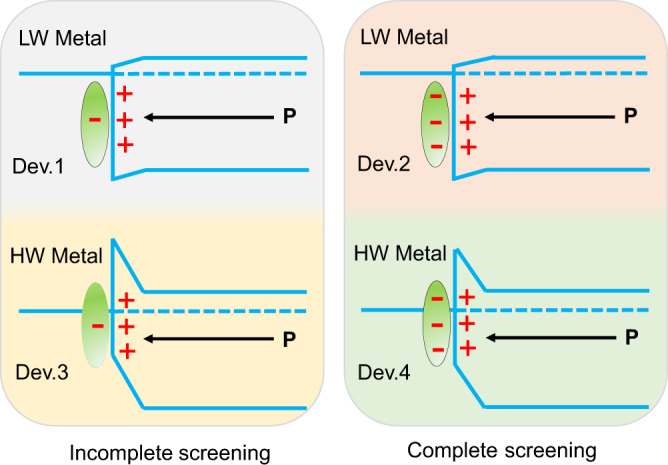
Band diagrams for presenting our approach to unravel the origin of ferroelectric resistance switching. A low-work-function (LW) metal will be employed to form a nearly zero interface barrier with the ferroelectric semiconductor (top left and top right), whereas a high-work-function (HW) metal will be used to obtain a relatively high barrier (bottom left and bottom right). Meanwhile, the interfacial screening of ferroelectric polarization charges is introduced into ferroelectric memristors: the left two panels indicate incomplete screening while those on right dictate complete screening. In total, four kinds of devices, termed Dev. 1, Dev. 2, Dev. 3, and Dev. 4, are created for this study. The black arrows in the band diagrams show polarization orientations. Both polarization charges and screening charges are illustrated at the right and left of interfaces, respectively.

To confirm the previously postulated FRS regime (i.e., polarization control over interface barrier), we split it into two critical aspects and separately control each of them—ferroelectric polarization and interface barrier—by interfacial engineering. First, we tentatively design specific α-In_2_Se_3_ ferroelectric memristors that are favorable to correlate FRS with interface barriers. In order to clearly probe Schottky barrier variation induced by polarization reversal, we will engineer the initial Schottky barriers of certain devices to a height of almost zero, as in Ohmic contact devices, by depositing low-work-function metals (top panel of Fig. 1). Meanwhile, relatively high, initial Schottky barriers will be also created using high-work-function metals, which helps to elucidate the effect of Schottky barriers on FRS (bottom panel in Fig. 1). Afterward, the correlative results acquired from both resistance switching and ferroelectric domain change could guide us to examine FRS with respect to Schottky barriers. Second, we plan to build another α-In_2_Se_3_ ferroelectric device aiming to associate FRS with ferroelectric polarization. Ferroelectric polarization can electrostatically induce polarization charges (i.e., bound charges) at interfaces, through which the FRS may occur. However, such polarization charges can be counterbalanced by either internally charged free carriers or externally charged molecules over the interfaces. For screening polarization charges, we intend to straightforwardly introduce charged external molecules into metal-ferroelectric interfaces. This approach will anticipatedly achieve both incomplete and complete screening of polarization charges as marked in the band diagrams (Fig. 1), and thereby may allow for the manipulation of the effect of ferroelectric polarization on FRS. Similarly, based on the resulting FRS behaviors and ferroelectric domain evolution, the contribution of ferroelectric polarization to FRS could be experimentally corroborated. In all, we attempt to unveil the FRS phenomenon by adopting four types of two-terminal α-In_2_Se_3_ ferroelectric devices, whose band diagrams are comparatively presented in Fig. 1. For simplicity, these devices are successively termed as Dev. 1, Dev. 2, Dev. 3, and Dev. 4.

**Fig. 2:**
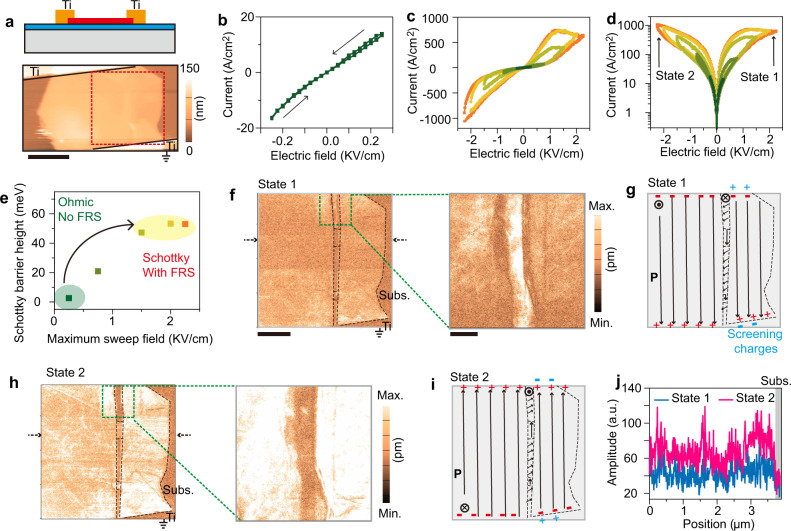
A ferroelectric memristor (Dev. 1) with almost zero primary interface barriers and incomplete screening effect. **a** Schematic cross-sectional structure (top) and atomic force microscopy (AFM) image of Dev. 1 (bottom). Low-work-function titanium (Ti) was utilized to form Ohmic contact with α-In_2_Se_3_. Note that the bottom electrode was grounded. Scale bar: 2 μm. **b** IV electrical curves of Dev.1 with electric field sweeping below the α-In_2_Se_3_ coercive field, indicating a good Ohmic contact (i.e., nearly zero barriers). The arrows show sweeping directions. **c** Linear and **d** logarithmic plots of IV electrical curves sweeping back and forth under different maximum electric fields. Colors from orange to green represent 2.25, 2.00, 1.5, 0.75, and 0.25 KV/cm, respectively. **e** Estimated interface barrier height as a function of maximum sweep electric fields. The actual height is an average value collected from five read biases (see Methods and Figure S3). **f** Piezoelectric force microscopy **(**PFM) amplitude image for the channel marked by red dashed lines (**a**) under state 1. The right shows the enlarged PFM mapping of the area highlighted by blue dashed lines in the left panel. Scale bars: 1 μm (left) and 0.2 μm (right). **g** Illustration of ferroelectric domain configuration in **f**. ${{{{{\boldsymbol{\otimes }}}}}}$ and $\odot$ represent out-of-plane polarization orientations pointing inwards and outwards across this paper, respectively, whereas the arrows show in-plane polarization directions. **h** PFM amplitude mapping of state 2 channel and (**i**) its domain configuration illustration. Screening charges are highlighted by blue in **g** and **i**. **j** Comparison of piezo-response amplitudes taken from the lines marked by blue arrows in **f** and red arrows in **h**, respectively. Gray indicates background substrate responses.

We start with an Ohmic contact α-In_2_Se_3_ ferroelectric memristor, i.e., Dev. 1. Its cross-sectional device structure is shown in the top panel of Fig. 2a, where low-work-function Ti was used to metalize a ferroelectric α-In_2_Se_3_ flake and form an expected initial Schottky barrier height of almost zero. The work functions of our used contact metals (i.e., Ti, Au, and Pt) are confirmed by ultraviolet photoelectron spectroscopy (Fig. S1) while related barrier heights are verified by Kelvin probe force microscopy (Fig. S2). As anticipated, Ti contact device exhibits the lowest Schottky barrier (i.e., 0) and Pt contact device reveals the highest barrier. During device fabrication, in addition to this interface metal, it is also important to acquire clean interfaces between metal and α-In_2_Se_3_ ferroelectrics to avoid too many charged residues absorbed or left at the interfaces, reducing the screening effect on polarization charges. The bottom of Fig. 2a presents the atomic force microscopy (AFM) image of Dev. 1. In this work, exfoliated α-In_2_Se_3_ flakes with 20–65 nm average thicknesses were employed to serve as channels in all devices. Fig. 2b shows IV electrical curves sweeping back and forth under small electric fields. Their straight IV character indicates Ohmic contact with desirable, initial barrier heights (i.e., nearly zero), whereas their lacking of electrical hysteresis manifests that swept electric fields are below the coercive field of ferroelectric α-In_2_Se_3_. When progressively raising the applied maximum electric field, the hysteresis in electrical curves (i.e., FRS) appears, expands and finally saturates with a greatest on/off ratio of 10, as shown in Fig. 2c and d. Their switching directions are explicitly labeled in Fig. S3a, where the current transitions for 1→2 and 3→4 can be attributed to the same changing trends in barrier heights for both terminals (Fig. S3b).

**Fig. 3:**
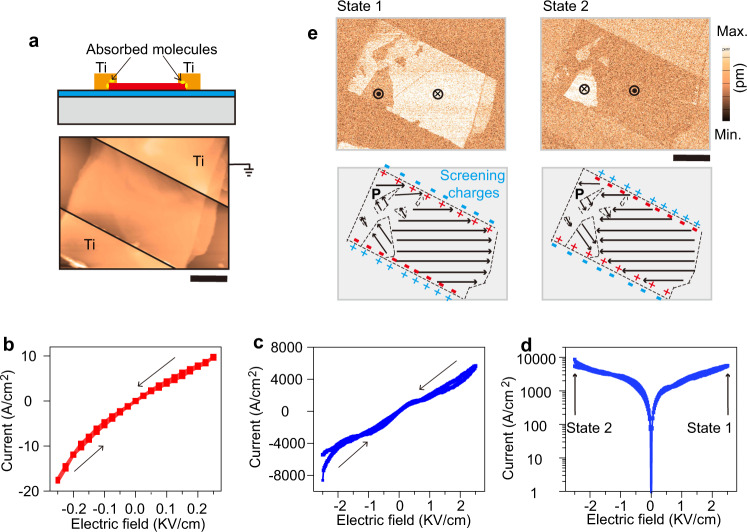
An Ohmic contact device (Dev. 2) with completely screened ferroelectric polarization charges at interfaces. **a** Cross-sectional illustration of Dev. 2 having charged molecules at interfaces (yellow ovals at the top), and AFM image of the practical device (bottom). IV electrical curves with sweeping back and forth under (**b**) small and (**c**) large electric fields. Note that the small electric field is below the ferroelectric coercive field. Sweeping directions are marked by the black arrows in **b** and **c**. **d** Logarithmic plot of **c**. **e** PFM amplitude mapping of state 1 and state 2. The bottom shows the schematics of corresponding ferroelectric domain conformations. Screening charges, as shown by blue, can fully compensate for polarization charges. Scale bar in **a** and **e**: 2 μm.

**Fig. 4:**
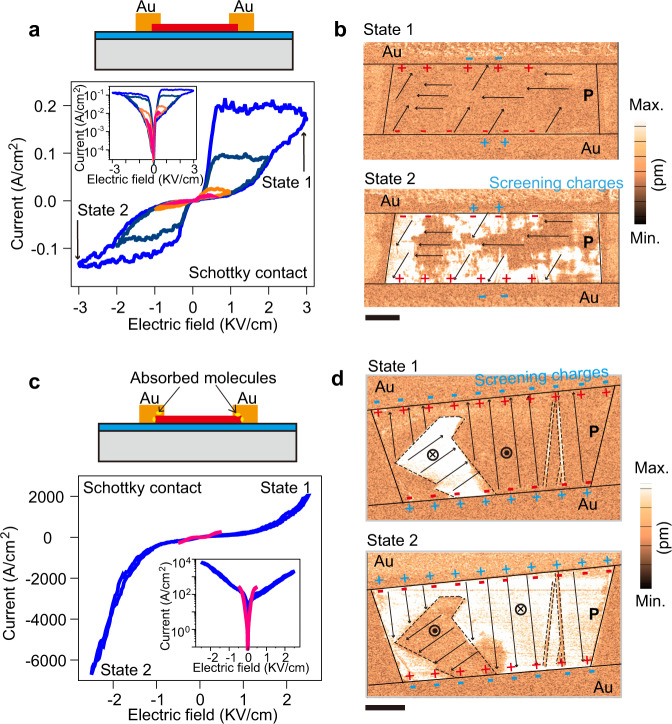
Schottky-contacted ferroelectric devices. **a** Cross-sectional illustration of Dev. 3 metalized by high-work-function gold (top panel; Schottky contact device), and its corresponding pinched hysteretic loops (bottom panel). The inset at the bottom shows a logarithmic plot of the loops. Colors from pink to blue indicate electric field sweeping of 0.5, 1, 2, and 3 KV/cm. **b** PFM amplitude mapping of Dev. 3 with state 1 and state 2. Scale bar: 500 nm. **c** A Schottky-contacted ferroelectric device with fully screened polarization charges (Dev. 4). The inset at the bottom panel shows electrical curves in logarithmic coordinates in a longitudinal direction. Red and blue curves represent 0.5 and 3 KV/cm sweeping. **d** PFM amplitude mapping of Dev. 4 for state 1 and state 2. Scale bar: 1 μm. For comparison, incompletely and completely screening charges are all labeled in **b** and **d** by blue.

When looking into the series of current switching curves (Fig. 2c), it is observed that the initial Ohmic contact of Dev.1 can gradually transform to Schottky contact as maximum electric fields increase, revealing an enhancement in Schottky barrier height. We extract corresponding changes in the barrier height under different maximum sweep fields through thermionic emission theory (see Methods and Fig. S3c). Their plots in Fig. 2e, together with the results in Fig. 2d, demonstrate that the occurrence of FRS must accompany the emergence of a relatively high Schottky barrier (i.e., non-zero value). The reproducibility of this observation can be confirmed by IV curves obtained from a separate device (Fig. S4). Importantly, this effect is challenging to detect and thus unusually ignored for ferroelectric memristors having a high, initial Schottky barrier, but is critical to design and optimize ferroelectric memristor performance. Moreover, the plots in Fig. 2e and S3c also suggest that, with the increase of sweep fields, the variation in Schottky barrier height becomes larger, giving rise to a great resistance switching ratio (Fig. 2d). After +2.25 KV/cm sweeping, the maximum barrier change is 53 meV, consistent with the value obtained by Kelvin probe force microscopy (Fig. S5; ~50 meV). A further illustration of FRS and Schottky barrier will be shown in Figs. 3 and 4. We note that, even though the signature of hysteresis evolution exhibited in Fig. 2c and d has been used to demonstrate the dominant role of ferroelectric polarization in FRS^6,8,21,22^, direct experimental evidence, such as piezoelectric force microscope (PFM) verified ferroelectric domain configuration, is still required.

Thus, we performed in situ PFM on Dev. 1. PFM response acquired from the entire channel is shown in Fig. S6a. For clearly observing domain evolution, we pay much attention to the PFM mapping of a specific area (i.e., red dashed lines in Fig. 2a and S6b), which contains multiple domains. Fig. 2f, h, S6c, and S6d display that, under poling state 1 (i.e., +2.25 KV/cm) and state 2 (i.e., −2.25 KV/cm), the resulting PFM amplitudes show remarkable differences in either in-plane or out-of-plane direction. Following our previous results^25^, these differences shall essentially result from whether out-of-plane α-In_2_Se_3_ dipole is aligned against or along the electric field of PFM scanning probe, and they suggest opposite orientations for state 1 and state 2 ferroelectric domains. Interestingly, multiple domains switching across Dev. 1 channel exists, which can be markedly manifested from enlarged PFM scanning images (right panels of Fig. 2f and h). These PFM results, along with intrinsic dipole orientations in α-In_2_Se_3_^26–28^ as interpreted in Fig. S7, enable us to draw concrete schematics of ferroelectric domain configurations for state 1 and state 2. Upon state 1, the majority of domains across the channel are aligned downward as a response to the applied electric field. Strip-like multiple domains are naturally embedded into the whole channel and most of them probably point to the left-bottom direction. As a result, positive and negative polarization charges electrostatically induce and reside in the bottom and top of Fig. 2g schematic. The incomplete screening of these polarization charges in Dev. 1 is also taken into account and is indicated by a few aggregated interfacial charges (i.e., blue). Besides the change in domain configuration in Fig. 2g, the device resistance is switched to a low value (read at, for example, +0.25 KV/cm) as well. After the application of an opposite poling field (i.e., state 2), relevant domain orientations, polarization charges, and screening charges, in principle, can swap signs as indicated in Fig. 2i. In addition to these variations, the device resistance is remarkably raised to a high value. Moreover, we derived PFM amplitudes from a given line marked by blue and red arrows, and plotted them in Fig. 2j for quantitative comparison. It can be seen that the state 2 amplitude is dramatically greater than that in state 1, proving the differences between their PFM amplitudes. Our PFM mapping above has unequivocally associated the FRS in Dev. 1 with ferroelectric polarization reversal. However, as previously reported^19,20,29,30^, nonferroelectric factors such as vacancy migration across the ferroelectric channel, accompanied by polarization switching, may still contribute to the FRS.

We then performed comparative experiments to further examine the exclusive influence of ferroelectric polarization reversal on FRS. The second type of α-In_2_Se_3_ ferroelectric memristor (Dev. 2) was conceived and fabricated (top panel of Fig. 3a) in which Ti still serves as the contact metal to create a nearly zero initial interface barrier as in Dev. 1. The significant discrepancy, compared to Dev. 1, lies in charged interface residues. By introducing external screening charges, α-In_2_Se_3_ ferroelectric polarization charges over Dev. 2 interfaces can be completely screened. If related electrical hysteretic loops expectedly disappear, vacancy migration across α-In_2_Se_3_ channel shall be firmly ruled out. Notably, upon fabricating the devices, charged photoresist residues and water molecules are good sources to bring external screening charges into the metal and ferroelectric interfaces. After lift-off, charged residues can be found on α-In_2_Se_3_ surfaces, particularly on nonuniform-thickness samples (Fig. S8a). These external charge centers inserted over the interfaces are illustratively shown by yellow ovals in the top panel of Fig. 3a. The bottom panel of Fig. 3a exhibits a typical AFM image of Dev. 2, where the channel is around 4 μm in length, larger than that in Dev. 1. We neglect inconsistent channel sizes because they have no notable influence on FRS phenomena. Fig. 3b, c and d show that under either small or large electric fields no obvious electrical hysteresis is seen, which contradicts with pinched loops in Fig. 1d. Nevertheless, as shown in Fig. 3e, S8b and c, PFM amplitude scanning demonstrates the presence of ferroelectric polarization reversal across the whole α-In_2_Se_3_ channel. Strikingly, several triangle-like ferroelectric domains are embedded in the channel, constituting a distinctive domain configuration. Furthermore, in the bottom panel of Fig. 3e we also show an illustration of ferroelectric polarization orientations for state 1 and state 2. It is emphasized that, despite the reversed signs of ferroelectric polarization charges (blue and red marks), the effect of ferroelectric polarization on FRS is indeed hidden by screening from external charges. These charges can be absorbed on both the top and side surfaces of α-In_2_Se_3_ channel owing to its large thickness (i.e., 60 nm), achieving complete screening as depicted in Fig. 1. Upon switching between state 1 and state 2, their polarization can be, though, flipped but their resulting device resistance remains almost at the same level, i.e., no FRS. Considering the absence of electrical hysteresis (i.e., no FRS) in Fig. 3b–d and the existence of hysteresis in Fig. 2c, we believe that vacancy migration within α-In_2_Se_3_ channel can be excluded. Overall, our interfacial engineering method has been proved to effectively tailor the screening level of ferroelectric polarization charges−incompleteness or completeness, which favors the examination of FRS origin. Taking all results in Figs. 2 and 3 together, we can demonstrate that FRS in our model system is a consequence of ferroelectric polarization reversal, and its induced Schottky barrier changes. We highlight that a relatively high barrier height is critical to producing FRS as well.

To give insight into FRS with respect to Schottky barriers, we replaced Ti with high-work-function Au (5.01 eV) and constructed a third type α-In_2_Se_3_ ferroelectric memristor (Dev. 3) as shown in Fig. 4a and S9a. Before metal deposition, cleaning and drying interfaces by plasma and gas, respectively, are pivotal to achieve incomplete screening. Note that these methods do not involve the reduction of interface defects and diffusion resulting from metal thermal evaporation. As exhibited in the bottom panel of Fig. 4a and Figure S9b, the nonlinearity of pinched hysteretic loops of Dev. 3 indicates a higher initial Schottky barrier formed at both terminals, compared with those of Dev.1 and Dev. 2. Consequently, its hysteretic windows become much larger and achieve a maximum on/off ratio of over 10^2^, consistent with our previous work^22^. The changing trend in pinched hysteretic loops reveals that the increase of maximum sweep field can lead to increased Schottky barrier variation and accordingly broadened switching windows. Another interesting fact for Dev. 3 is that its current switching differs from that of Dev.1 owing to the modulation of polarization charges with opposite signs (Fig. S9c and d). Moreover, regarding state 1 and state 2 in Dev. 3, we also visualized corresponding ferroelectric domain configurations (Fig. 4b) by PFM. Irregular shape domains, for example, those indicated by dark areas across the α-In_2_Se_3_ channel in state 2, are detected. Upon applying ±3 KV/cm electric fields, such domains remain almost intact owing to their orientation perpendicular to the poling field. Despite this, ferroelectric polarization charges over the interfaces can still switch their signs due to the flipping of bright-area domains, rendering channel resistance switching between a small value from state 1 and a large value from state 2 (read at a positive field). To elaborate on the related incomplete screening effect, a few external charges are indicated by blue in the bottom schematics of Fig. 4b. We note that out-of-plane PFM amplitude mapping for Dev. 3 shows no obvious responses probably because of the intrinsic odd-even effect in ferroelectricity (see Fig. S9e for more details). Fig. 4a and b demonstrate that a high, initial Schottky barrier is indispensable to produce a large FRS ratio likely through suppressing off current. To verify this, on and off currents extracted from a read field with the greatest switching ratio are presented in Fig. S10, where 19 Schottky devices and 15 Ohmic devices are involved. Schottky devices generally exhibit larger switching ratios, proving again that a high initial Schottky barrier plays a significant role in obtaining optimal FRS. In addition, in the case of complete screening by external charges, no marked FRS is observed in Schottky contact devices (Dev. 4) as shown in Fig. 4c, whereas its ferroelectric polarization switching is still pronounced (Fig. 4d and S11). Albeit different domain configurations in state 1 and state 2, their device resistances, read at any small sweep fields, are nearly overlapped. This coincides with the observation from Dev. 2 with an Ohmic contact, which rules out other intertwined mechanisms such as vacancy migration and further validates the vital impact of ferroelectric polarization reversal on FRS.

Our systematic studies of Dev. 1–4 suggest that the FRS in our α-In_2_Se_3_ model system intrinsically originates from ferroelectric polarization reversal and its related change in the Schottky barrier, and can be tuned by the initial Schottky barrier height as well. Most previous works^6,8,9,21^ have attributed FRS to polarization control over interface barriers, which may complicate the relationship between polarization and interface barriers and thus lead readers to intuitively believe that the interface barrier is strictly contingent on polarization reversal. We note that those three are independent variables, none of which can be dispensable for rationally optimizing FRS. Although electrostatically induced ferroelectric charges (i.e., polarization charges) at interfaces directly give rise to a change in Schottky barrier height, these electrostatic charges are naturally prone to be screened by charged free carriers and externally absorbed molecules. Taking the variability of these screening charges into account, polarization-charge-triggered Schottky barrier change can be deemed as the second variable, apart from ferroelectric polarization. Furthermore, a feasible barrier height is a requirement for the emergence of FRS, and therefore it can also be believed as the third variable.

**Fig. 5:**
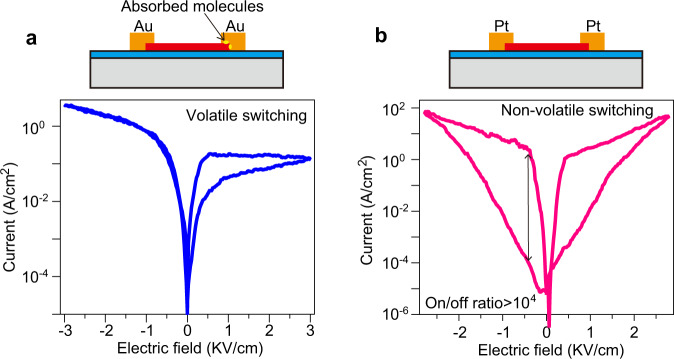
Engineered interfaces for functional memory devices. **a** IV curves (bottom panel) for a volatile ferroelectric memristor are achieved by introducing screening charges with different quantities into two terminals as shown in the top panel. **b** IV curves for a nonvolatile memristor with a high on/off ratio, which is realized by the deposition of high-work function metal onto ferroelectric α-In_2_Se_3_. A great on/off switching ratio over 10^4^ is demonstrated as marked by the arrow.

Having established the underlying FRS regime in α-In_2_Se_3_ memristors and discovered its three variables, we next designed functional and competitive devices. By using externally charged molecules, the effective manipulation of the impact of ferroelectric polarization charges on FRS, as described in Fig. 3, inspires us to create two disparate interfaces: one holds incompletely screened polarization charges while the other has fully screened polarization charges. Such a design guarantees the successful implementation of volatile ferroelectric α-In_2_Se_3_ memristors. Its representative IV curves in Fig. 5a show that, as the sweep field reaches +3 KV/cm, a high resistance state incrementally transitions to a low resistance state. However, upon negative electric field sweeping, the overlapped curves manifest that this low resistance state cannot be continuously maintained, which constitutes a volatile property and distinguishes nonvolatile memristors. These volatile ferroelectric memristors that feature an abrupt decay of historical device conductance when powered off can serve as basic circuit components for building ferroelectric neuromorphic computing systems^31^. In addition, through adopting high-work-function Pt (5.06 eV) as a contact metal to raise initial Schottky barriers, ferroelectric memristors with large on/off switching ratios (over 10^4^) can be successfully obtained (Fig. 5b). Although Pt interface barrier is higher than Au barrier (Fig. S1), the current density for Pt contact device, for example, read at +3 KV/cm, is three orders of magnitude greater than that in Au counterpart. Such an abnormal behavior likely stems from the impact of interface roughness scattering on electron transport due to the large thickness variations over α-In_2_Se_3_ surfaces of Pt device. The volatile and nonvolatile devices presented in Fig. 5 can be seamlessly combined by using just one α-In_2_Se_3_ fake and can substitute selector and memristor stacking in common neural circuits for creating vdW ferroelectric based computing hardware.

## Discussion

We stress that the perfect screening (i.e., no current switching windows) as in Dev. 2 and Dev. 4 is not caused by large currents, for example, above 2000 A/cm^2^. Regarding our four types of α-In_2_Se_3_ ferroelectric devices, their intrinsic carrier concentration, though, involves interior screening to ferroelectric polarization charges but can be assumed to be constant, delivering the same screening effect from interior carriers for all devices. The discrepancies of contact areas, other than carrier concentration, are essentially responsible for great current inconsistencies between different devices. Despite the presence of large channel currents even above 2000 A/cm^2^, remarkable current switching windows can be still discerned for those devices without the intentional introduction of exterior screening charges (Fig. S12). This suggests an incomplete screening of ferroelectric polarization charges over the α-In_2_Se_3_ metal interface systems, which contrasts the perfect screening in Dev. 2 and Dev. 4. Thus, the contribution of interior free carriers to the screening effect is limited. Moreover, interfacial defects and diffusion produced by the bombardment of high-energy metal atoms on α-In_2_Se_3_ surfaces (Fig. S13) may serve as another source of screening charges. However, it can be also disregarded owing to the identical conditions of metal evaporation for all devices. Therefore, it is emphasized that exterior charges as fulfilled in Dev. 2 and Dev. 4 are predominant to screen ferroelectric polarization charges, making current switching windows adjustable. In addition, we note that the presence of ferroelectric polarization charges can simultaneously build an internal electric field across ferroelectric materials, called the depolarization field. Previous studies^32–35^ have reported that this field has a detrimental effect on the stability of ferroelectric polarization that is the origin of polarization charges, and on the retention property of as-fabricated memristors. To contend with this field (which can be also termed as a polarization-charge field), external screening charges are highly required. However, considering the critical role of polarization charges in tuning FRS, a tradeoff must be finely made between screening charges and polarization charges for achieving desirable device performance, which needs future exploration.

In conclusion, we have proposed an interfacial engineering approach to unravel the FRS origin in ferroelectric memristors. Through the interfacial engineering of α-In_2_Se_3_ ferroelectric memristors, the screening effect of polarization charges, and interface barriers can be tailored, which provides an opportunity to take all FRS elements into account. We find that FRS is indeed determined by three independent variables: ferroelectric polarization, Schottky barrier change, and initial barrier height, as opposed to traditional interpretation. Based on these findings, functional volatile ferroelectric memristors and large-switching-ratio ferroelectric memristors are demonstrated, which paves the way for building compact neutral circuits by connecting the selector and memristor in a cell using just one α-In_2_Se_3_ flake.

Our interfacial engineering approach can also apply to other ferroelectric materials-based ultrashort-channel or long-channel memristors to understand their FRS origin. This interfacial engineering allows that three types of specific devices can be successfully fabricated. The first one is an ideal Ohmic contact device with a zero barrier; the second one still has an Ohmic contact but with an externally charged residue layer inserted over the interfaces; the third one is a Schottky contact device with a higher barrier. By observing their hysteretic IV curves and concurrent domain configurations as demonstrated above, the ferroelectricity origin can be strongly confirmed.

## Methods

### Device fabrication and measurements

The α-In_2_Se_3_ flakes were mechanically exfoliated from the parent crystal. E-beam lithography was used to pattern α-In_2_Se_3_ memristor electrodes. After pattern lithography and subsequent development, cleaning electrode areas particularly those on α-In_2_Se_3_ surfaces, is critical to decrease the introduction of charged residues and restrain the screening to polarization charges. To remove polymer residues on patterned electrode areas, a Plasma Striper with a mixture gas of O_2_ and Ar was used to treat α-In_2_Se_3_ surfaces. The mass flow of O_2_ and Ar were set to 100 sccm and 14 sccm, respectively. The chamber pressure, power density, and treatment time were 0.5 mTorr, 350 W, and 60 s, respectively. Then, a nitrogen spray gun was used to dry the patterned electrode areas for 2 minutes to remove water molecules. Next, electron beam evaporation (i.e., Denton Vacuum Explorer) was used to deposit Ti and Au films. The chamber vacuum was pumped to 5E-6 Torr. A slow evaporation speed of 0.5 Å/s was adopted to obtain high-quality crystalline Au and Ti films. For Dev. 1 and Dev. 2, 30-nm Ti was chosen to act as the Ohmic contact metal. For Dev. 3 and Dev. 4, we first evaporated 3-nm Ti as an adhesive layer and then deposited 70-nm Au to serve as the contact metal. Note that such a thin layer of Ti would not form a uniform film and impact the modulation of high-work-function Au on the interface barrier. In addition, Equipment Support Company Sputtering system was used to sputter 70-nm Pt films with a 1 Å/s deposition rate using DC mode.

Electrical measurements were performed in a Keithley 4200 Parameter Analyzer, while AFM and PFM were carried out using Asylum MFP-3D with a 2 N/m probe. During PFM measurements, dual AC resonance mode with scan biases of 0.5–0.8 V was used to detect ferroelectric electromechanical responses. UPS measurement was conducted in Kratos AXIS Ultra DLD with the incident photon energy of 21.22 eV.

### Estimation of Schottky barrier height

We estimate Schottky barrier variation over the interfaces from a simplified equation, which is extracted from the thermionic emission and diffusion theory.

1 $${{{{{\rm{In}}}}}}[{I}_{{{{{{\rm{HRS}}}}}}/}{I}_{{{{{{\rm{LRS}}}}}}}] \sim -\varDelta {\varnothing }_{r}/KT$$

where *I*_HRS_ and *I*_LRS_ represent the currents of high resistance state and low resistance state, respectively, and −$\triangle {\varnothing }_{r}$ is the reverse-biased barrier. The barrier changes with respect to reading biases and maximum sweep fields for Dev. 1 can be derived from IV hysteretic loops (Fig. S3c). Because the initial barrier height of Dev. 1 can be regarded as 0, the actual barrier height after the application of maximum sweep fields is roughly equal to the corresponding barrier variation. Thus, the actual Schottky barrier height as a function of maximum sweep fields can be obtained as exhibited in Fig. 2e.

## Supplementary information

Supplementary information file

## Data Availability

All other data supporting this study are available from the corresponding authors upon reasonable request. Source data are provided with this paper.

## Source data

Source data
