# Supplementary material for: Unraveling the origin of ferroelectric resistance switching through the interfacial engineering of layered ferroelectric-metal junctions
Source: Nat Commun. 2021 Dec 15;12:7291. doi: 10.1038/s41467-021-27617-6 (PMC8674284; doi:10.1038/s41467-021-27617-6)
Supplement: Supplementary file 1 — Supplementary information file [file 41467_2021_27617_MOESM1_ESM.pdf]

Supplementary information

Unraveling the origin of ferroelectric resistance switching through the interfacial engineering of layered ferroelectric-metal junctions

Fei Xue[1,2#*], Xin He[2#], Yinchang Ma[2#], Dongxing Zheng[2], Chenhui Zhang[2], Lain-Jong Li[2], Jr-Hau He[3], Bin Yu[1], and Xixiang Zhang[2*]

[1]Hangzhou Global Scientific and Technological Innovation Centre, Zhejiang University, Hangzhou 311200, China.

[2]Physical Science and Engineering Division, King Abdullah University of Science and Technology, Thuwal 23955-6900, Saudi Arabia.

[3]Department of Materials Science and Engineering, City University of Hong Kong, Kowloon, Hong Kong, China.

[#]These authors contributed equally.

*Corresponding authors: fei.xue@kaust.edu.sa; xixiang.zhang@kaust.edu.sa

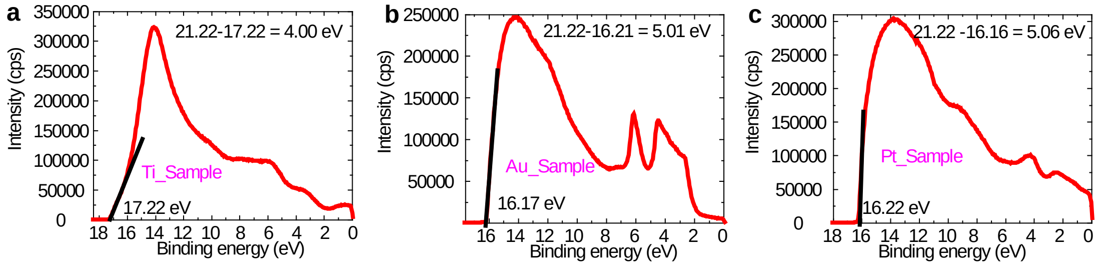

Figure S1. **UPS measurements to determine metal work functions**. The results acquired from Ti, Au and Pt samples are successively shown in **(a)**, **(b)** and **(c)**. The work functions are calculated by the following equation: $q\emptyset_f = hv$-W, where $q\emptyset_f$ indicates the work function, hv dictates the incident phonon energy, and W is the energy cut-off. As marked in these plots, the actual work functions for Ti, Au, Pt films are 4.00 eV, 5.01 eV, and 5.06 eV, respectively.

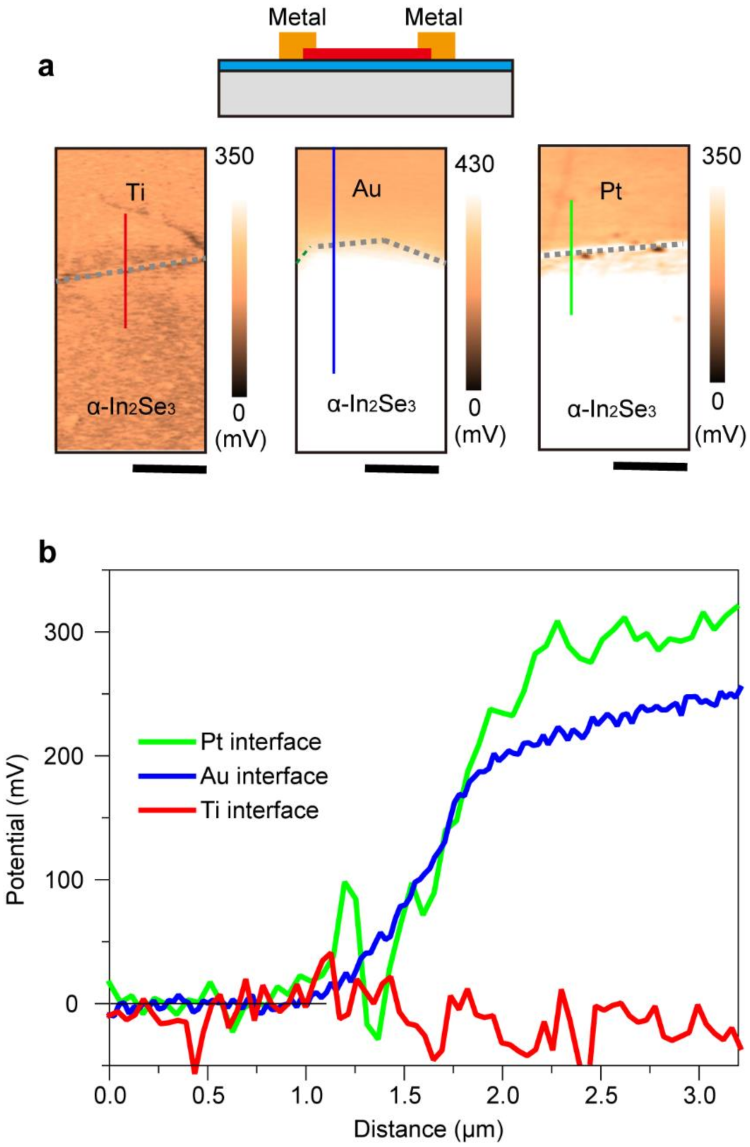

Figure S2. **Measuring the surface potential differences of different α-In_2Se_3-metal interfaces**. **(a)** KPFM surface potential mapping collected from different contact metals, i.e., Ti, Au and Pt. Scale bar: 2 μm for Ti and Pt interfaces; 1 μm for Au interface. **(b)** Quantitative surface potential differences extracted from corresponding lines in (a). Multiplying these surface potential differences by e can be used to estimate the corresponding Schottky barrier height ($q\emptyset_b$).

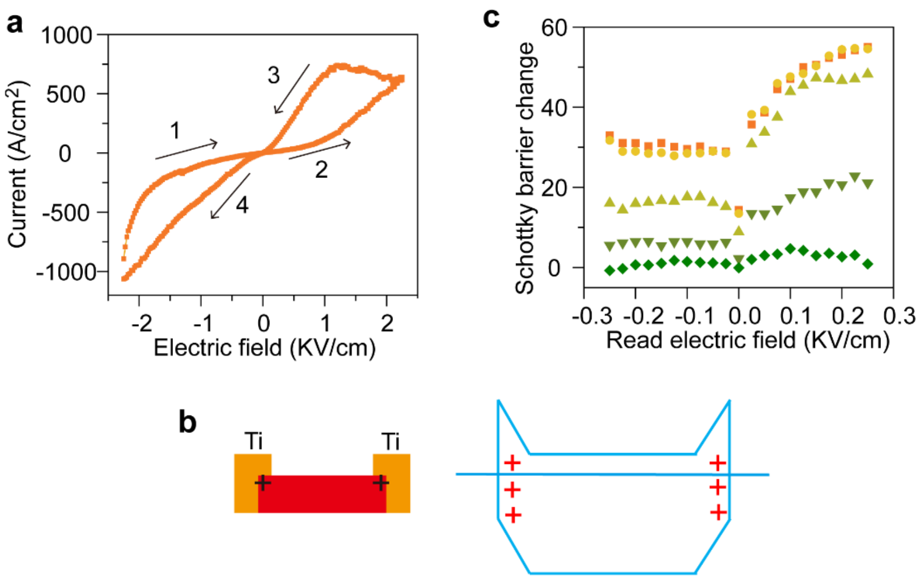

Figure S3 (a) IV electrical curves reproduced from Figure 2c in the main text for exhibiting the directions of resistance switching. **(b)** Proposed band diagram for the observed switching behaviors in (a). Over the two interfaces, ferroelectric polarization charges hold the same sign, resulting in transitions 1→2 and 3→4. **(c)** Extracted Schottky barrier change as a function of small read electric fields. The different colors represent different maximum sweep fields, which are in line with Figure 2c.

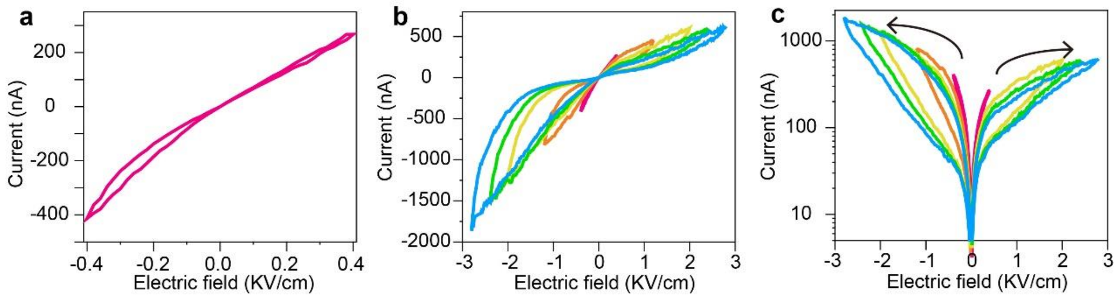

Figure S4. Electrical curves collected from a separate device that is similar to the Dev. 1 structure. (a) IV curves sweeping back and forth under small electric fields. This linear character suggests the formation of Ohmic contact with a almost zero barrier. **(b)** Pinched hysteretic loops with different maximum electric fields. **(c)** Logarithmic plot of the curves in (b). It is seen that, with increasing maximum electric fields, hysteretic windows are gradually opened and IV curves are bent, revealing a barrier transition from almost zero to a high value.

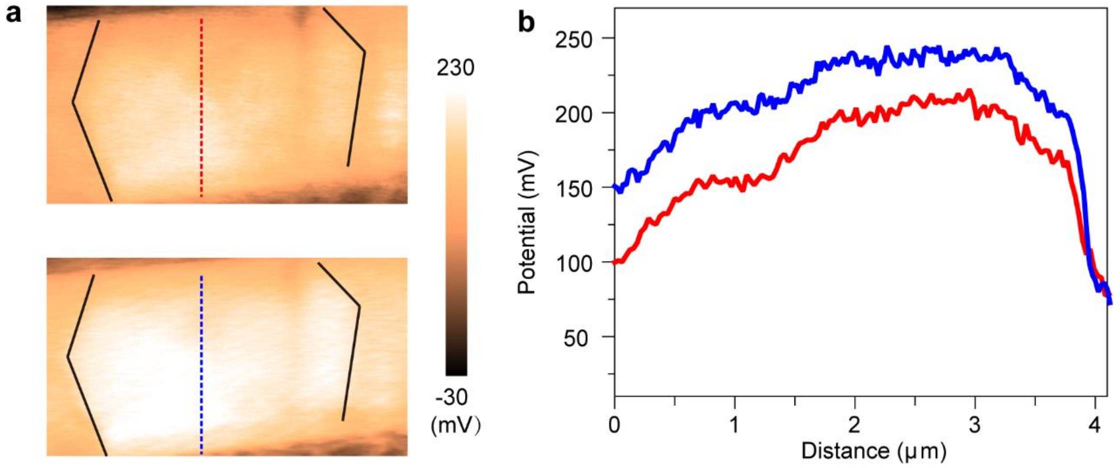

Figure S5. **Surface potential mapping of Dev. 1**. **(a)** KPFM surface potential mapping of Dev. 1. The device channel is highlighted by black lines. The top and bottom indicate potential images under state 1 and state 2, respectively. **(b)** Potential differences with different poling voltage, i.e., +2.25 KV/cm for red and -2.25 KV/cm for blue. These data are taken from the dashed lines in (a).

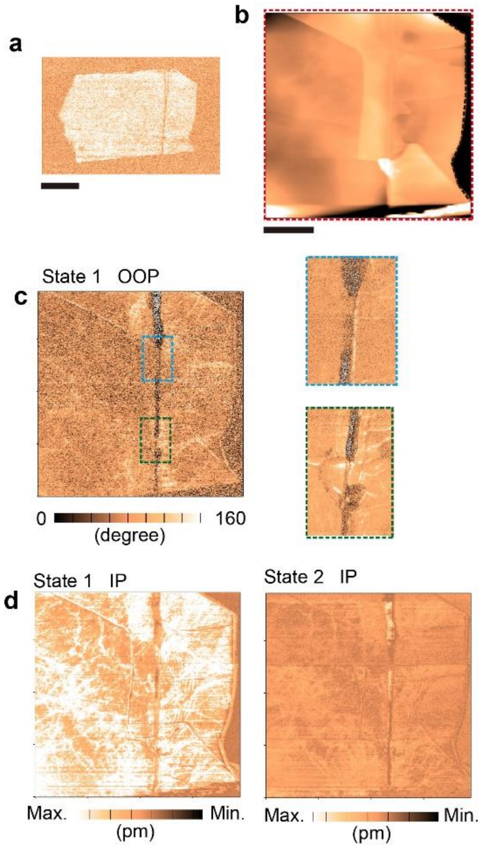

Figure S6 (a) PFM amplitude mapping of the whole channel of Dev. 1. Scale bar: 2 μm. **(b)** AFM topography image of the outlined area in Figure 2a. Scale bar: 1 μm. **(c)** Out-of-plane PFM phase mapping under state 1. The two small images on the right show the corresponding enlarged areas in the left panel as outlined by blue and green. **(d)** In-plane PFM amplitude mapping of Dev. 1.

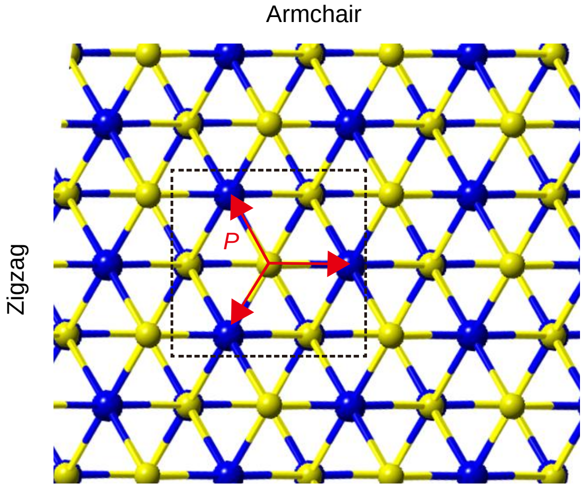

Figure S7. Schematic α-In_2Se_3 crystal structure taken along z axis (blue: In atom, yellow: Se atom). The horizontal and vertical directions indicate armchair and zigzag edges, respectively. Red arrows mark the three types of in-plane α-In_2Se_3 dipole along [100], [010] and $[\bar{1}\bar{1}0]$ directions.

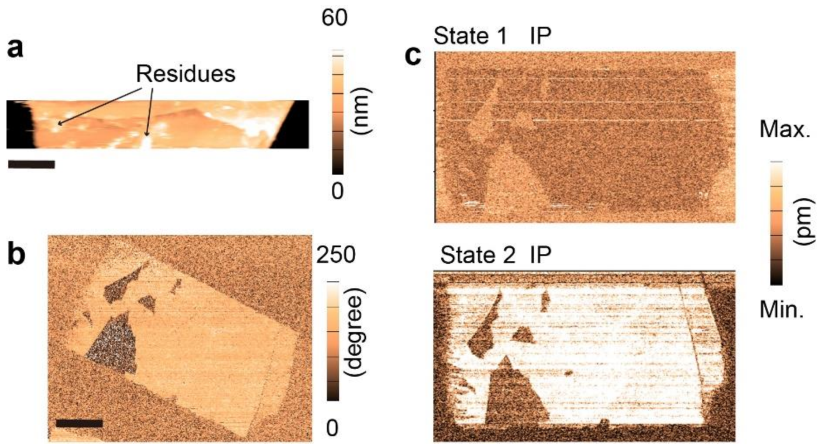

Figure S8 (a) AFM topography image of a typical α-In_2Se_3 surface showing the surface contamination by charged residues. Scale bar: 2 μm. **(b)** PFM phase mapping of the device. Scale bar: 2 μm. **(c)** In-plane PFM amplitudes in state 1 and state 2.

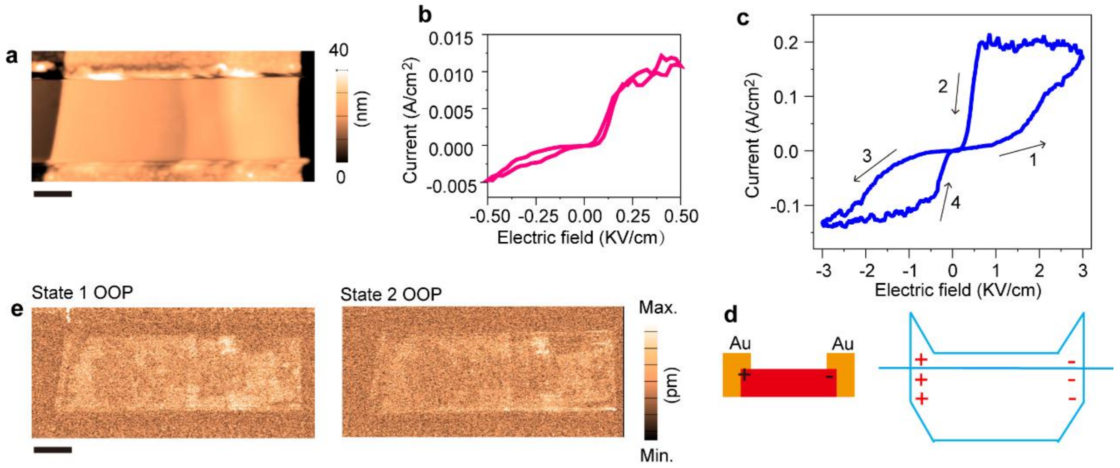

Figure S9 (a) AFM image of Dev. 3. Scale bar: 1μm. **(b)** Enlarged IV curves for the pink in Figure 4a. **(c)** IV curves for presenting the switching directions. **(d)** Proposed band diagram model for explaining the switching directions. **(e)** Out-of-plane PFM amplitude mapping. Scale bar: 500 nm.

As previously reported[1], ferroelectricity in hexagonal α-In_2Se_3 exists in odd layers but cancels out in even layers due to the unique atomic stacking. Most exfoliated α-In_2Se_3 flakes exhibit nonuniform thicknesses like the channel samples of Dev. 1, Dev. 2 and Dev. 4. The α-In_2Se_3 thickness differences across the channel provide the possibility to simultaneously generate in-plane and out-of-plane ferroelectricity. However, in the case of identical thicknesses in both terminals, the out-of-plane ferroelectricity may disappear, which can be revealed by the PFM amplitude in Figure S9e.

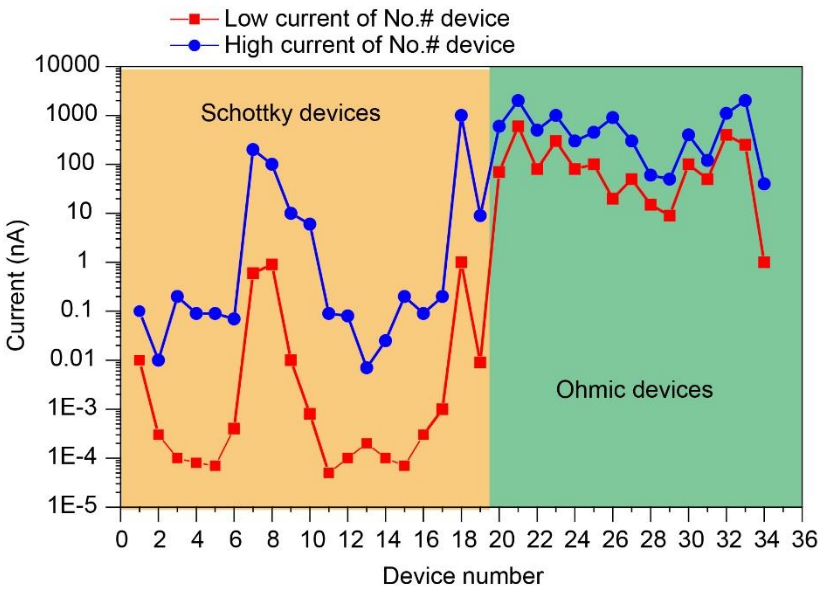

Figure S10 Statistic data of on and off switching currents for Schottky and Ohmic devices.

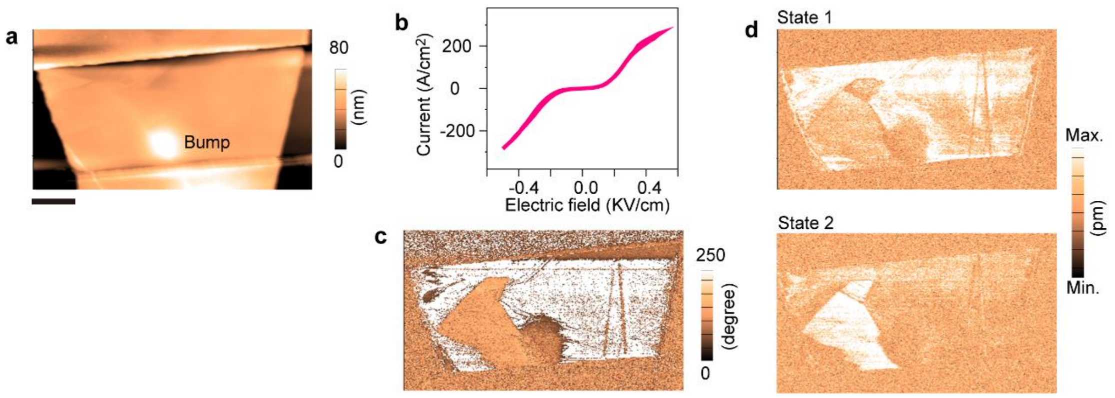

Figure S11 (a) AFM topography image of the device in Figure 4b (Dev. 4). The bump within the channel is possibly caused by substrate nanoparticles. Scale bar: 1 μm. **(b)** IV electrical curves under small electric field sweeping. The data is reproduced from Figure 4c, showing a good Schottky contact. **(c)** PFM phase mapping of the device. **(d)** In-plane PFM amplitude mapping.

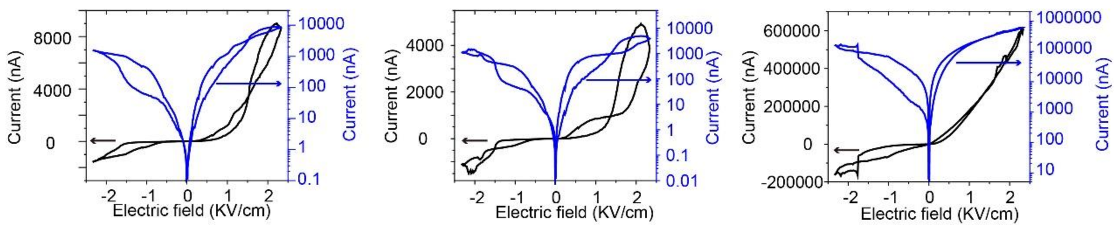

Figure S12 IV curves from three representative ferroelectric memristors with switching windows but large currents. The fabricating conditions are the same as those of Dev. 4.

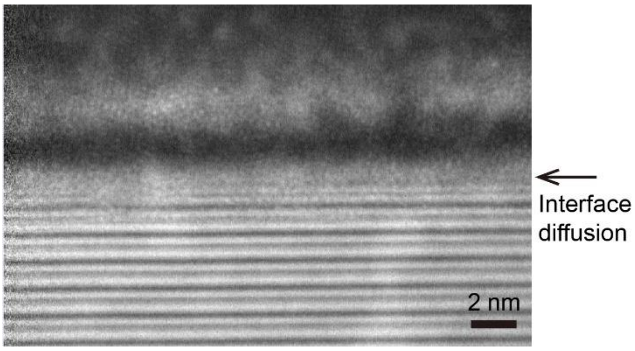

Figure S13 High-resolution cross-sectional transmission electron microscopy image of Dev. 2. The interface diffusion can be observed across the metal α-In_2Se_3 interface.

Reference

1. Baohua Lv, Zhi Yan, Wuhong Xue, Ruilong Yang, Jiayi Li, Wenjuan Ci, Ruixue Pang, Gang Liu, Peng Zhou, Zhongyuan Liu, Wenguang Zhu and Xiaohong Xu. Layer-Dependent Ferroelectricity in 2H-Stacked Few-Layer α-In_2Se_3. ***Materials Horizons***, 8, 1472-1480 (2021).
